# Prevalence and risk factors of *M tuberculosis* infection in young people across 14 communities in Zambia and South Africa

**DOI:** 10.1371/journal.pgph.0002077

**Published:** 2023-09-29

**Authors:** Modupe Amofa-Sekyi, Ab Schaap, Linda Mureithi, Barry Kosloff, Maina Cheeba, Bxyn Kangololo, Redwaan Vermaak, Robynn Paulsen, Maria Ruperez, Sian Floyd, Petra de Haas, Sarah Fidler, Richard Hayes, Helen Ayles, Kwame Shanaube, on behalf of the TREATS study team

**Affiliations:** 1 Zambart, University of Zambia School of Medicine, Lusaka, Zambia; 2 Department of Infectious and Tropical Disease Epidemiology, London School of Hygiene & Tropical Medicine, London, United Kingdom; 3 Health Systems Research Unit, Health Systems Trust, Cape Town, South Africa; 4 Clinical Research Department, London School of Hygiene and Tropical Medicine, London, United Kingdom; 5 KNCV Tuberculosis Foundation, The Hague, Netherlands; 6 HIV Trials Unit, Imperial College London, London, United Kingdom; Faculty of Medicine, Khon Kaen University, THAILAND

## Abstract

**Background:**

From 2018–2021 the TB Reduction through Expanded Antiretroviral Treatment and TB Screening (TREATS) project took place in 21 Zambian and South African communities. The TREATS Incidence of TB Infection Cohort Study was conducted in adolescents and young people (AYP), aged 15–24 years in 14 communities. We describe the baseline prevalence and risk factors of *Mycobacterium tuberculosis* (*M*. *tuberculosis)* infection among this cohort and explore the quantitative QFT-Plus interferon gamma (IFN-γ) responses.

**Methods and findings:**

A random sample of approximately 300 AYP per community were recruited and information on TB/HIV risk factors, TB symptoms and social mixing patterns collected. QuantiFERON TB Gold Plus assay (QFT-Plus) was used to detect *M*. *tuberculosis* infection, following manufacturer’s instructions. Logistic regression was used to determine factors associated with infection. 5577 eligible AYP were invited to participate across both countries, with 4648 enrolled. QFT-Plus results were available for 4529: 2552(Zambia) and 1977(South Africa). Overall, 47.6% (2156/4529) AYP had positive QFT-Plus results, the prevalence of infection in South Africa being twice that in Zambia (64.7% (1280/1977) vs 34.3% (867/2552) p<0.001). Infection was associated with age, household contact with TB and alcohol in Zambia but showed no associations in South Africa. The antigen tube differential (TB2-TB1>0.6 IU/ml) of the assay at baseline showed no evidence of association with recent TB exposure.

**Conclusion:**

The high prevalence of infection in AYP warrants urgent action to address TB control, especially in South Africa. Further research is required to delineate antigen tube responses of the QFT-Plus assay more precisely to fully realise the benefit of the additional TB2 tube in high TB/HIV burden settings.

## Introduction

Tuberculosis (TB) is one of the leading causes of death globally from a single infectious agent, *Mycobacterium tuberculosis* (*M*. *tuberculosis*) [1]. A quarter of the world’s population is estimated to be infected with *M*. *tuberculosis*. Those infected have an estimated 5–10% lifetime risk of progression to active TB disease in the general population, however among people living with HIV this risk is 10% annually [2]. The synergistic effect of the TB/HIV epidemics has led to the Southern African region having the highest rates of TB globally with TB incidence rates of 554/100,000 per year in South Africa and 319/100,000 per year in Zambia [3]. The TB disease burden in those aged 10–24 years has been challenging to quantify until recently, as routine TB data were reported for children aged ≤ 14 years and adults aged ≥15 years [4]. A global estimate from a modelling exercise in 2012 showed 1.8 million adolescents and young people (AYP) develop the disease annually accounting for 17% of the overall TB burden [5].

AYP are a key group at a uniquely important phase of development. The impact of adolescent hormonal changes on the immune response to pathogens including *M*. *tuberculosis* and modifications in numbers and functions of leucocytes, may affect their progress from infection to disease [6, 7]. Traditionally TB infection rates have been measured in young children but their measurement in AYP may provide a useful gauge of TB transmission in the community [8, 9].

With no gold standard test to detect infection, current tests measure the immune response to *M*. *tuberculosis* antigens. A limitation of these tests is their lack of ability to differentiate which individuals are recently infected and more likely to progress to disease, from those who were remotely infected. A fourth generation IGRA, the QuantiFERON-TB Gold Plus assay (QFT-Plus), (Qiagen, Hilden, Germany), includes two *M*. *tuberculosis* specific antigen tubes (TB1 and TB2) as well as positive and negative controls(nil and mitogen tubes) [10]. The TB1 and TB2 contain ESAT-6 and CFP-10-derived long peptides that stimulate CD4-mediated interferon gamma response. Additionally, the TB2 tube contains shorter peptides stimulating CD8-mediated responses. It has been suggested that the tube differential between interferon gamma (IFN-γ) responses of the TB2 and TB1 tubes (TB2 minus TB1) may therefore reflect CD8-mediated activity [11]. As CD8-mediated activity has been associated with recent exposure, it is suggested that QFT-Plus may have a role in detecting acute infection [12, 13]. A comparative contact tracing study done in Italy suggested that QFT-Plus may be as accurate as and show stronger associations with surrogate measures of TB exposure than the previous IGRA QuantiFERON-TB Gold in Tube (QGIT) [14]. It has also been suggested that this test may perform better than other tests in HIV-infected individuals who may have a low CD4 count, with a recent study in Zambia, showing that the overall sensitivity of QFT-Plus was not affected by HIV status [15].

From 2018–2021 the TB Reduction through Expanded Antiretroviral Treatment and TB Screening (TREATS) project was carried out in 21 Zambian and South African communities which had been part of the combined TB/HIV intervention HPTN071 (PopART) trial [16–18]. TREATS was conducted to measure the effect of the PopART intervention on the burden of TB in these communities. The TREATS Incidence of TB Infection Cohort Study (TREATS Infection Cohort) was conducted in AYP, aged 15–24 years. It offered an opportunity to better understand the prevalence of infection in AYP and QFT-Plus responses. This cohort was followed up for 2 years to measure the incidence of infection. The main aim of this report is to describe the baseline prevalence and risk factors of infection among this cohort.

## Methods

### Study design and setting

This paper reports the baseline analysis of a cohort study conducted in 14 urban communities in Zambia (8 communities) and the Western Cape, South Africa (6 communities). These communities had been part of the HPTN 071 (PopART) trial, a cluster randomized trial of universal testing and treatment for HIV, with a primary outcome of HIV incidence(16). One of the TB outcomes measured by TREATS was the incidence of TB infection among AYP (TB infection cohort) in 7 intervention and 7 standard of care communities (Arm A and C communities of the PopART trial respectively).

During the HPTN 071 (PopART) trial an enumeration process was conducted in 2013 with the location of each household in the community recorded. This provided a sampling frame for the selection of households for the TREATS infection cohort. Each community was subdivided into 10 equally sized zones and each zone was further divided into blocks of approximately 40 households in Zambia and 55 in South Africa. To ensure adequate geographical representation, a two-stage sampling process was applied: (1) zones were put in a random order and (2) one block of households was randomly selected from each zone. The 10 selected blocks were visited sequentially. All households within a selected block were eligible and approached until the target of 300 consenting AYP per community was reached. In case the target was not met after visiting the first ten blocks of households, new blocks were randomly selected from the same zones by repeating the second stage of sampling described above.

All household members were enumerated and eligible AYP invited to a health facility (Zambia) or clinical research site (South Africa) for enrolment. Inclusion criteria were age 15–24 years, usually resident in the community for at least 2 years previously, intention to stay in the community for the next 2 years, and ability to give informed consent. The exclusion criteria were being on TB treatment and participation in TB vaccine trials or other TB prevention trials. Recruitment occurred from July 2018-May 2019, with follow up for 24 months.

### Study procedures

Written voluntary informed consent for all study procedures was obtained for eligible participants aged 18 years and above at the clinical research site/health facility. Parental consent and participant assent were obtained for participants aged 15–17 years. Password-protected tablets were used by trained research assistants to record information on socio-demographic data, TB/HIV history, known TB risk factors, and social mixing patterns of the AYP, via the administration of a structured questionnaire. Multiple questions were used to enquire about social spaces frequented by the AYP within the past week, as a proxy for social mixing patterns.

A TB symptom screen (continuous cough >2 weeks, fever > 1-week, unintentional weight loss, drenching night sweats, chest pain, haemoptysis, shortness of breath) was conducted. Symptomatic participants provided a sputum sample for Xpert MTB/RIF Ultra (Cepheid, Sunnyvale, CA, United States). Height and weight were recorded. Blood samples were taken for QFT-Plus testing and rapid HIV testing was offered to all.

Xpert-Ultra positive participants were referred to the TB clinic for care and excluded from the cohort. QFT-Plus positive and/or HIV positive participants were counselled and referred for appropriate care: TB preventative therapy and/or ART as per national guidelines. Participants were informed of the signs and symptoms of TB and asked to report for further investigations at the local health facility if any developed.

### Laboratory procedures

#### QFT-Plus procedure

We collected 12 mls of blood in two 6ml lithium heparin tubes at the study site which were transferred to a laboratory within 6 hours for QFT-Plus testing. 1ml of the heparinized blood was aliquoted into each of the QFT-Plus incubation tubes (total 4 ml) and incubated for 24 hours as per manufacturer’s protocol. In 6/8 communities in Zambia the incubation step was conducted at nearby regional laboratories. In 2/8 Zambian communities, the incubation step was performed at the nearby Central Research Laboratory (CRL). After incubation, each incubation tube was centrifuged. Using different sterile pipettes for each of the four tubes the plasma was transferred into matching colour-coded storage tubes and stored at -20C to -80C. These frozen samples, stored at the four regional laboratories were transferred in portable -20C freezers to the CRL in Lusaka at monthly intervals and stored at -20C to -80C until used for testing. Samples from all 8 communities were tested in batches at the CRL by enzyme linked immunosorbent assay (ELISA) as per manufacturer’s instructions. In South Africa, the heparinized blood from all communities were transported to a central laboratory in Cape Town for the incubation step and the post-incubation plasma aliquots were transported to a centralised laboratory in Johannesburg for ELISA testing. In both countries the QFT-Plus Analysis Software was used to calculate results. In Zambia, rapid HIV testing was also conducted at the laboratory for those without a HIV test result from the study site.

### Definitions

#### *M*. *tuberculosis* infection

An overall positive QFT-Plus response, defined as per manufacturer’s instructions, was taken as proxy for infection. The IFN-γ response was calculated by subtracting the IFN-γ concentration of the nil tube from that of the TB1 or TB2 tubes (TB1-nil or TB2-nil), with the antigen tube with the higher IFN-γ concentration being taken as the test result. For a QFT-Plus result to be valid the response of the antigen tubes must have been > 25% of the negative control. An overall positive QFT-Plus result was defined according to the manufacturer’s cut point as an IFN-γ concentration ≥ 0.35 IU/ml. An indeterminate result occurs with high nil >0.8 IU/ml or low mitogen <5.0 IU/ml concentrations.

#### Household contact

A household contact (HHC) for TB was defined as member of the participant’s household who was currently on TB treatment or who had previously been on TB treatment.

#### Recent exposure to TB

Recent exposure to TB was defined as having a household contact currently on TB treatment.

### Statistical analysis

Our current analysis is restricted to the baseline measurement of infection and to participants with a valid QFT-Plus result. The analysis was done separately by country due to the known differences in TB epidemiology.

With a planned sample size of 4200 AYP (300 per community) and assuming 100 (2.4%) AYP were recently exposed to TB, with 4100 AYP not recently exposed, our study would have 93% power to detect a two-fold increase in the odds of being QFT-positive among recently exposed AYP.

Exposure variables analysed for their association with QFT-Plus positivity included HHC, age, sex, alcohol use, HIV status, smoking amongst others.

A random effects logistic regression model with household as the panel variable (to control for household clustering) was used to calculate odd ratios adjusted for age, sex and community between each exposure variable and QFT-Plus positivity. The p value was calculated using the likelihood ratio test comparing the regression model with and without the exposure variable. The effect was additionally adjusted for any other variable found to be associated with QFT-Plus positivity (with p<0.1) after adjusting for age, sex and community. The household contact variable was included in the final adjusted model irrespective of association in the first adjusted model, as household contact was deemed the most important potential risk factor for infection. We note that since the outcome (infection) is a common event, the odds ratio may differ appreciably from the risk ratio in these analyses.

Concordance between the antigen tube IFN-γ responses was evaluated using the kappa statistic. Restricting our analyses to those with a positive QFT-Plus, we described the IFN-γ response in the TB1 tube(TB1-nil) and the TB2 tube (TB2-nil) as a continuous variable amongst the different strata of household exposure. The signed-rank test was used to describe differences in mean and median TB1 and TB2 IFN-γ responses among different strata of HHCs. We also explored binary QFT-Plus antigen tube positivity among AYP with different types of HHCs. Binary antigen tube positivity was denoted as TB1+TB2+ when there was a positive IFN-γ response on both TB1 and TB2 tubes. TB1+TB2- was used to denote a TB1-only positivity. TB1-TB2+ was used to denote a TB2-only positivity.

We hypothesized that:

1. AYP recently exposed to TB would show greater IFN-γ responses in the TB2 tube compared to AYP exposed to TB in the past
2. The IFN-γ response of antigen tube differential (TB2-TB1) would indicate the CD8-mediated response of assay and hence be associated with recent exposure.

We evaluated the hypotheses using a logistic regression model adjusted for age, sex, and community. To account for intrinsic test variability a (TB2-TB1)> 0.6 IU/ml was taken as a positive CD8-mediated response as described by Metcalf et al. [19]. Data were analysed using STATA, version 16.0 (StataCorp LP, College Station, TX, USA).

### Ethical considerations

Ethical approval for the study was obtained from the ethics committees of the University of Zambia (UNZABREC), the London School of Hygiene and Tropical Medicine, and the Pharma -Ethics Research Committee in South Africa.

## Results

**Fig 1:**
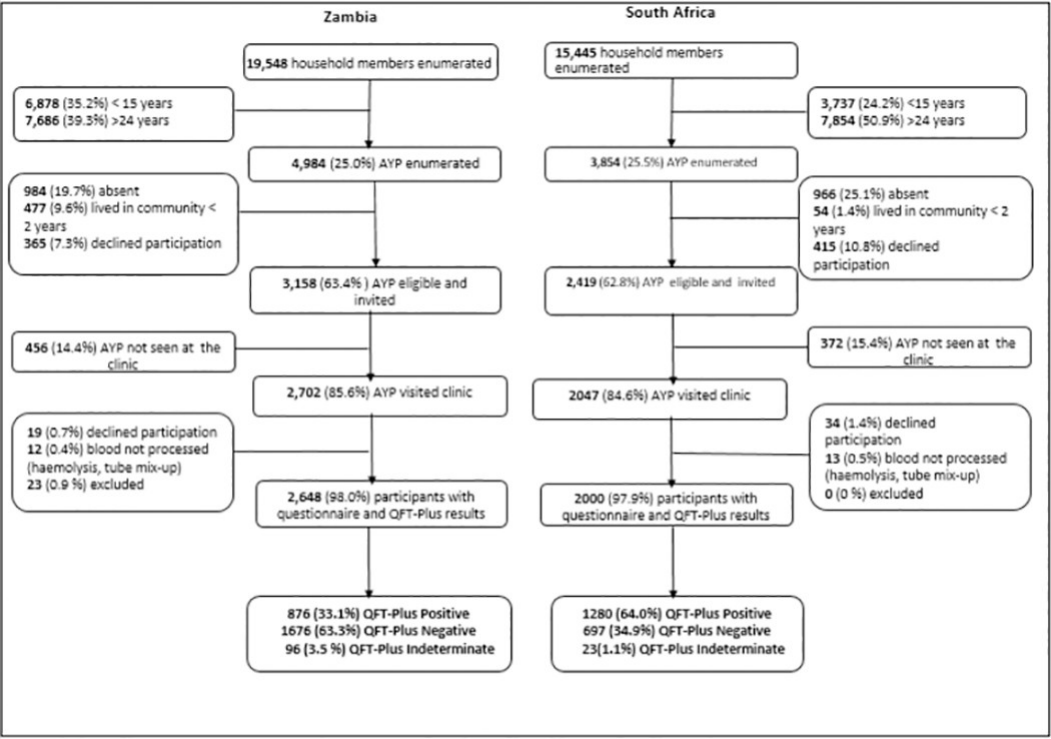
Flow chart of study participants.

A total of 5577 AYP were eligible and invited to participate in the study across both countries (3158 from Zambia and 2419 from South Africa). In both countries approximately 85% of the invited participants visited the study site with 98% consenting to the study, and about 98.0% providing questionnaire responses and a blood sample. A total of 4648 AYP were enrolled into the cohort (Fig 1).

QFT-Plus results were available for 4529 participants, 2552-Zambia and 1977- South Africa, excluding participants with indeterminate results. The demographic characteristics of the cohort are presented in Table 1.

**Table 1:** Demographic characteristics and HIV status of the cohort.

Characteristic	ZAMBIA			SA		
	N	n	% Column	N	n	% Column
**Overall**	2552			1977		
**Sex**						
Male		1138	44.6%		860	43.5%
Female		1414	55.4%		1117	56.5%
**Age group**						
15–16		573	22.5%		473	23.9%
17–18		566	22.2%		408	20.6%
19–20		536	21.0%		398	20.1%
21–22		478	18.7%		352	17.8%
23–24		399	15.6%		346	17.5%
**HIV Status** *						
HIV Negative		2418	94.8%		1155	58.4%
HIV Positive		129	5.1%		51	4.2%

* HIV positivity rates in the table are restricted to those with a known HIV result with missing values for individuals with an unknown HIV status 5/2552 in Zambia and 711/1977 in South Africa not shown

In Zambia 55.4% (1414/2522) and in SA 56.5% (1117/1977) of the cohort were female. In both countries the median age was 19.5 years (IQR 17.2–22.0). Of those with a known HIV status, HIV positivity was 5.1% (129/2552) in Zambia and 4.2% (51/1977) in South Africa. In South Africa 39.0% (771/1977) of the cohort had an unknown HIV status as they did not report on their HIV status and declined to test at the research site.

### QFT-Plus positivity

Overall, 47.6% (2156/4529) of the cohort across both countries had positive QFT-Plus results, however, the prevalence among AYP in South Africa was twice that of Zambia (64.7% (1280/1977) vs 34.3% (876/2552), p< 0.001) (Tables 2 and 3).

**Table 2:** QFT-Plus positivity risk factor analysis-Zambia*.

Characteristic	N	% Column	QFT-Plus Positive	% (row)	AOR 1(95%CI)	p	AOR 2^Z^ (95%CI)	p
**Overall**	2552		876	34.3%	0.46 (0.41–0.52)			
**Sex**						0.922		0.295
Male	1138	44.6%	381	33.5%	1		1	
Female	1414	55.4%	495	35.0%	0.99 (0.81–1.20)		1.12 (0.91–1.37)	
**Age group**						<0.001		0.001
15–16	573	22.5%	160	27.9%	1		1	
17–18	566	22.2%	178	31.5%	1.17 (0.87–1.56)		1.11 (0.83–1.49)	
19–20	536	21.0%	171	31.9%	1.19 (0.88–1.60)		1.73 (0.79–1.45)	
21–22	478	18.7%	186	38.9%	1.66(1.23–2.24)		1.43 (1.04–1.94)	
23–24	399	15.6%	181	45.4%	2.29 (1.67–3.15)		1.89 (1.37–2.62)	
**HIV Status** **						0.389		0.332
HIV Negative	2418	94.8%	827	34.2%	1		1	
HIV Positive	129	5.1%	46	35.7%	0.91 (0.58–1.42)		0.88 (0.56–1.37)	
**Household contacts (HHC)** ***						0.011		0.021
No HHC	2201	86.3%	725	32.9%	1		1	
HHC^1^	256	10.0%	113	44.1%	1.47 (1.08–2.01)		1.42 (1.04–1.94)	
HHC^2^	41	1.6%	18	43.9%	1.60 (0.76–3.33)		1.67 (0.80–3.48)	
HHC^3^	28	1.1%	14	50.0%	2.71 (1.11–6.61)		2.51 (1.02–6.13)	
**Smoking status**						0.020		0.543
Non- Smoker	2259	88.5%	748	33.1%	1		1	
Ex-smoker	172	6.7%	75	43.6%	1.57 (1.06–2.31)		1.25 (0.83–1.87)	
Current smoker	121	4.7%	53	43.8%	1.58 (1.01–2.48)		1.13 (0.70–1.84)	
**Alcohol Intake**						0.001		0.014
Never	1995	78.2%	629	31.5%	1		1	
Monthly	255	10.0%	108	42.4%	1.35 (0.99–1.87)		1.28 (0.92–1.77)	
2–4 times a month	48	9.72%	112	45.2%	1.85 (1.32–2.58)		1.73 (1.21–2.46)	
5 or more times a month	54	2.1%	27	50.0%	2.00 (1.04–3.84)		1.76 (0.89–3.45)	

HHC1: Household contacts with past history of TB, HHC2: Household contacts on current TB Treatment, HHC3: Household contacts with past history of TB and current TB Treatment

AOR 1: Adjusted odds ratio: analysis adjusted for age, sex and community

AOR 2^Z^: Adjusted odds ratio: analysis adjusted for age, sex and community, household contact, smoking and alcohol use

*Analysis excludes indeterminate QFT-plus results

**Missing values 5/2552 for HIV status not shown

*** Missing values 26/2552 (1.0%) for HHC not shown

**Table 3:** QFT-Plus positivity and risk factor analysis-South Africa*.

Characteristic	N	% (Column)	QFT plus Positive	% (row)	AOR 1 (95%CI)	p	AOR 2^SA^ (95%CI)	p
**Overall**	1977		1280	64.7%	1.92 (1.72–2.15)			
**Sex**						0.086		0.198
Male	860	43.5%	575	66.9%	1		1	
Female	1117	56.5%	705	63.1%	0.83 (0.67–1.03)		0.88 (0.71–1.10)	
**Age group**						0.018		0.068
15–16	473	23.9%	275	58.1%	1		1	
17–18	408	20.6%	260	63.7%	1.26 (0.94–1.70)		1.23 (0.90–1.65)	
19–20	398	20.1%	271	68.1%	1.49 (1.09–2.03)		1.43 (1.01–1.95	
21–22	352	17.8%	242	68.8%	1.63 (1.19–2.26)		1.53 (1.10–2.11)	
23–24	346	17.5%	232	67.1%	1.47 (1.06–2.03)		1.37 (0.99–1.90)	
**HIV Status** **						0.182		0.133
HIV Negative	1155	95.5%	763	66.1%	1		1	
HIV Positive	51	4.2%	29	56.9%	0.66 (0.35–1.25)		0.60 (0.32–1.14)	
**Household contacts (HHC)** ***						0.336		0.400
No HHC	1670	84.5%	1066	63.8%	1		1	
HHC^1^	214	10.8%	151	70.6%	1.28 (0.91–1.81)		1.26 (0.89–1.78)	
HHC^2^	45	2.3%	32	71.1%	1.28 (0.64–2.59)		1.27 (0.63–2.55)	
HHC^3^	38	1.9%	27	71.1%	1.52 (0.70–3.27)		1.47 (0.68–3.16)	
**Smoking status**						0.079		0.102
Non- Smoker	1331	67.3%	823	61.8%	1		1	
Ex-smoker	22	1.1%	12	54.6%	0.70 (0.28–1.76)		0.70 (0.28–1.74)	
Current smoker	624	31.6%	445	71.3%	1.30 (1.01–1.67)		1.28 (1.00–1.65)	
**Alcohol Intake**						0.603		0.702
Never	1087	55.0%	684	62.9%	1		1	
Monthly	503	25.4%	323	64.2%	0.92 (0.71–1.18)		0.94 (0.73–1.21)	
2–4 times a month	299	15.1%	210	70.2%	1.11 (0.81–1.52)		1.08 (0.78–1.40)	
5 or more times a month	88	4.5%	63	71.6%	1.23 (0.72–2.11)		1.12 (0.65–1.92)	

HHC1: Household contacts with past history of TB, HHC2: Household contacts currently on TB Treatment, HHC3: Household contacts with past history of TB and currently on TB Treatment

AOR 1: Adjusted odds ratio: analysis adjusted for age, sex and community

AOR 2^SA^: Adjusted odds ratio: analysis adjusted for age, sex and community, household contact and smoking

*Analysis excludes indeterminate QFT-Plus result

**Missing values 771/1977 for HIV status not shown

*** Missing values 10/1977 (0.5%) for HHC not shown

### Risk factors for infection among AYP

In Zambia, age, having a household contact and alcohol showed evidence of association with infection. The 23–24 age group were more likely to be infected than the 15–16 age group [aOR 1.89 95% CI 1.37–2.62, p = 0.001]. There was evidence of an association between having a household contact and infection, p = 0.021. Compared to having no HHC, the adjusted odds ratio (aOR) in those with a HHC with TB in the past was [aOR 1.42 95%CI 1.04–1.94], for those having a HHC with current TB was aOR 1.67 (95%CI(0.08–3.48) and for those with HHCs with both past and current TB was aOR 2.51 (95%Cl 1.02–6.13). Compared to those who reported no alcohol consumption, participants reporting an alcohol consumption of 2–4 times a month were more likely to be infected aOR 1.73 (95%CI 1.21–2.46) than those who reported a monthly consumption aOR 1.28 (95% CI 0.92–1.77) or those who reporting alcohol consumption >5 times a month aOR 1.76 (95% CI 0.89–3.45 (Table 2).

In South Africa, none of the variables showed evidence of an association with infection. Additionally, no associations were noted between infection and household density or number of people sharing a room in both countries (S1 and S2 Tables).

#### Antigen tube IFN-γ responses

Overall, 44.6% (2018/4529) of participants were positive on both TB1 and TB2, 1.2% (54/4529) on TB1 alone, and 1.9% (84/4529) on TB2 alone. There was 97.0% concordance between positivity of TB1 and TB2 tubes of QFT -Plus according to the manufacturer’s algorithms (kappa = 0.94, p<0.001).

### Recent exposure to TB and CD8-mediated responses

**Fig 2:**
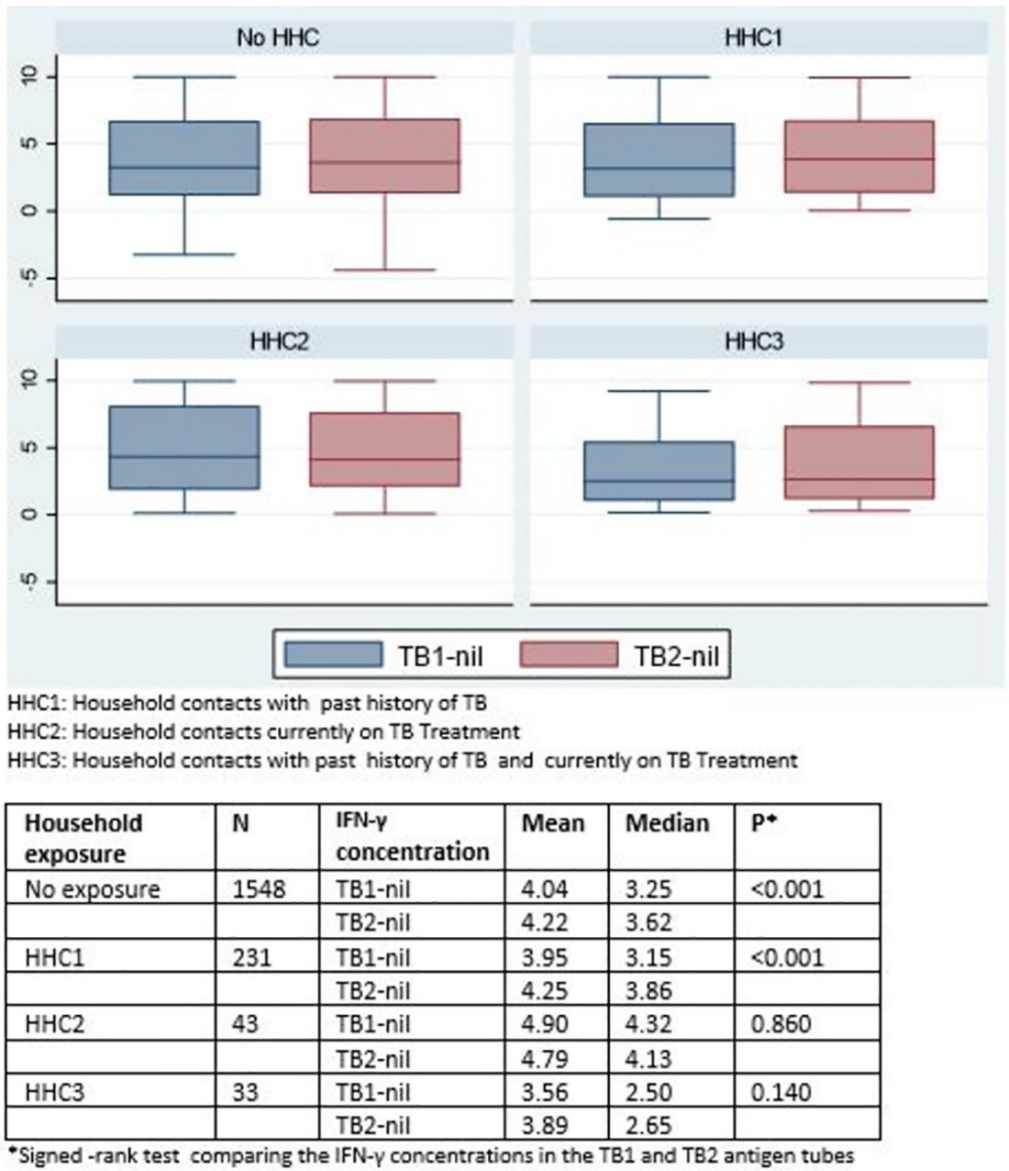
Box plot of IFN-γ concentrations of the TB1 and TB2 antigen tubes among different strata of household exposure.

There were higher mean (4.25 IU/ml) and median (3.86 IU/ml) TB2 tube IFN-γ responses compared to that of the TB1 IFN-γ tube responses (mean 3.95IU/ml, median 3.15IU/ml), p<0.001 among those with a HHC for previous TB treatment. A similar trend was noted in individuals with no household contacts with mean (4.22 IU/ml) and median (3.62 IU/ml) TB2 IFN-γ responses higher than TB1 tube IFN-γ responses (mean 4.04 IU/ml, median 3.35IU/ml, p<0.001). We did not find differences between TB1 and TB2 IFN-γ responses among those reporting HHC with current TB (Fig 2).

Compared to having no HHC, participants reporting a HHC with TB in the past were more likely to have a TB2-only response [aOR 2.12 95%CI 1.15–3.90, p = 0.016]. No evidence of association was observed between reporting a HHC with current TB and a TB2-only response [aOR 0.60 95%CI 0.08–4.61, p = 0.621]. There was no evidence of association between reporting a HHC with both past and current TB and a TB2-only response [aOR 0.67 95%CI 0.09–5.26, p = 0.707] (Table 4). There was no evidence of an association between any of these household contact categories and a tube differential response reflecting CD8- mediated activity (Table 4).

**Table 4:** Analysis of CD8 mediated IFN-γ responses among household contact categories.

HHCs categories*	Participants with QFT-Plus tube positivity **	Participants positive on TB2 tube only (TB1-TB2+)	n/N (%)	Adjusted OR (95%CI)	p	TB2-TB1> 0.6 IU/ml	n/N (%)	Adjusted OR (95%CI)	p
	N	n				n			
**Total**	2156	84	4.7			394	18.3		
**No HHC**	1791	63	3.5	1		323	18.0	1	
**HHC** ^**1**^	246	18	6.8	2.12 (1.15–3.90)	0.016	50	18.9	1.25 (0.86–1.82)	0.242
**HHC** ^**2**^	50	1	2.0	0.60 (0.08–4.61)	0.621	9	18.0	1.19 (0.53–2.68)	0.667
**HHC** ^**3**^	41	1	2.4	0.67 (0.09–5.26)	0.707	10	24.4	1.82 (0.86–1.82)	0.149
**missing**	10	1	10.0	-	-	2	20.0	-	-

HHC1: Household contacts with past history of TB

HHC2: Household contacts currently on TB Treatment

HHC3: Household contacts with past history of TB and currently on TB Treatment

TB1-TB2+: positive only on TB2 but not TB1

Adjusted OR: analysis adjusted for age, sex and community

*Missing values 10/2156 for tube positivity 10/2156 not shown

**Participants with positive QFT-plus result expressed as composite tube positives **TB1+TB2+ /TB1+TB2-/ TB1-TB2+**

## Discussion

We described the baseline prevalence of, and risk factors associated with *M*. *tuberculosis* infection determined by the QFT-Plus assay in a cohort of adolescents and young people in a Zambian and South African cohort.

### Performance of the QFT-Plus assay

Several studies have investigated the performance of QFT-Plus and other TB infection diagnostics. A study evaluated the accuracy of QFT-Plus compared to QGIT in Italy among 179 individuals with TB infection diagnosed by QGIT, active TB patients, individuals cured of TB (defined as individuals who had completed 6 months of treatment after culture-confirmed TB and who were culture negative after treatment) and healthy controls [20]. The authors found the two assays to have similar sensitivity and specificity. The sensitivity of QFT-Plus in those with active was TB 90% compared to 88% with QGIT. The specificity of both QFT-Plus and QGIT was 100% in healthy controls.

Likewise, a study among 989 low-risk healthcare workers (HCW) in the United States showed comparable performance between QFT-Plus and QGIT [21]. The agreement between the QFT-Plus and QGIT was reported as 95.6% (95%CI 94.3–96.9, kappa 0.57). Among 626/989 HCW with no risk-factors and no self-reported history of TB infection, QFT-Plus positivity 3.0%(CI 1.7–4.3) was greater than QGIT positivity 2.1% (CI 1.0–3.2, p = 0.24) with no evidence of a difference in results the two tests.

A study was carried out among pregnant women in Kenya to estimate prevalence of TB infection and the effect of HIV infection on performance of QFT-Plus in comparison to TST [22]. In this study 400 women (200 HIV-infected and 200 without HIV infection) were enrolled. Both groups of women had similar overall QFT-Plus positivity 31.5% in HIV- infected compared to 33.2% women without HIV infection. The diagnostic yield of infection was found to be three-fold with QFT-Plus (32%) compared to TST(12%). The was some evidence of a difference between the median individual antigen tube responses among the HIV infected compared to the HIV uninfected women; TB1(1.05 vs. 2.65 IU/ml, p = 0.035) and TB2 (1.26 vs. 2.56IU/mL, p = 0.027).

### Prevalence of *M*. *tuberculosis* infection

While the overall prevalence of infection, measured by QFT-Plus positivity was 47.6%, AYP in South Africa had twice as much infection (64.7%) as compared to Zambia (34.3%). The Western Cape Province, South Africa generally has one of the highest TB burdens in the world and our results reflect this [23, 24]. A previous study carried out in Zambia and South Africa among adult HHC of TB patients found high levels of infection measured by QGIT(63.6%) and TST (40.2%) [25]. When stratified by country the infection prevalence measured by TST was higher in South Africa (50% 95% CI 24–77%) than Zambia (31% 95%CI 7–73%). Another study carried out in the same province in South Africa among adolescents aged 12–18 years reported prevalence measured by QGIT as 50.9% [26]. A recent study among adolescents (10-19yrs) in rural Kwazulu Natal, South Africa found a prevalence of 23.0% using QFT-Plus [9]. The presence older AYP in our cohort may partially explain the higher prevalence of infection seen compared to the previous studies metioned. Our finding of double the prevalence of infection in South Africa as compared to Zambia is in keeping with the pattern of disease prevalence in both countries. When the cohort was being recruited a TB symptom screen was used to indentify individuals with presumptive TB. This may have caused us to miss some instances of subclinical TB which has been shown to contribute signifcantly to the TB burden [27]. A recent analysis the 2013–2014 Zambian National TB prevalence survey data showed that about 40.5% with bacteriologically confirmed TB were asymptomatic for cough ≥2 weeks [28]. In a cross-sectional survey in South Africa between 2017–2019, 57.5% of bacteriologically confirmed TB occurred in those who were asymptomatic but had chest Xray abnormalities [29].

### Risk factors

In Zambia, the association of age with infection may reflect the cumulative exposure of the older AYP to TB and therefore the greater risk of infection. The association of infection with household contact with past or present TB cases in Zambia points to household transmission still playing an important role in transmission of TB. The association of alcohol intake with infection in Zambia may be explained by alcohol use leading to impaired immune function which may lead to increased susceptibility to infection, while increased alcohol use may also be accompanied by social behavioral patterns leading to greater exposure to TB [30, 31].

In South Africa the observed lack of association between infection and household contacts with TB cases, has been seen in a previous study in Cape Town, South Africa which compared the relationship between household exposure to TB and infection among children and adolescents [32]. The authors found prevalent infection was positively associated with household exposure in children aged 5–14 years but found no association in adolescents >15 years. It is possible with the high TB burden in South Africa that community transmission would overshadow any household transmission in the AYP. Social contact patterns have been examined and modelled in these communities through a detailed social contact survey among adults over a 24- hour recall period as reported by Dodd et al. [33]. They found most close contacts occurred at home with casual contacts occurring at church, shops, in the interviewee’s work building, and in other homes. A recent systematic review on social contact patterns revealed that in lower income countries, AYP have similar mixing patterns to other age groups [34]. Irrespective of where the exposure to TB occurs, we generally expect the older adults who have had a greater cumulative exposure over time to have a greater burden of infection, compared to the younger individuals like children. In our data we see this trend reflected in both countries with the 23–24 age group showing a higher percentage of infection (Zambia-45.4%, South Africa 67.1%) compared to the 15–16 age group (Zambia-27.9%, South Africa- 58.1%) (Tables 2 and 3) with the trend being more pronounced in Zambia compared to South Africa.

### Household exposure and antigen tube responses

We hypothesized that individuals with a HHC currently on TB treatment, are recently exposed and therefore would show greater TB2-only positivity (TB2+TB1- expression) because of CD8-mediated immune responses. We analysed this hypothesis in two ways.

Firstly, we looked for an association between being recently exposed and having a TB2-only response compared with past TB exposure. Secondly, we looked at CD8-mediated activity by comparing the tube differentials in recent exposure and past exposure.

We observed that individuals with a HHC with TB in the past twice as likely to have a TB2-only response compared to those recently exposed. We found no association between CD8-mediated activity and recent exposure.

This contrasts with findings from low burden TB settings. An Italian study carried out among 119 HHC found CD8-mediated activity was associated with sleeping in proximity to the index TB case [14]. A large QFT-Plus verification laboratory-based study in Netherlands and Belgium examining QFT-Plus test results for patients who had QFT-Plus testing for different indications found that individuals tested because of contact investigation and periodic checks by occupational health services were more likely to have greater CD8-mediated activity as compared to individuals tested for different indications [35]. The authors considered the contact investigation testing group to be more recently infected. In contrast, a multi-centre study conducted in 19 centres in 6 European and 1 South American country found that CD8-mediated activity was not associated with recent exposure [36].

To the best of our knowledge our study is the first where the antigen tube differential has been explored for associations with recent exposure in high TB-HIV burden settings. The lack of association in our study between recent exposure and CD8-mediated activity may be explained in part by several factors. It is challenging to define when and by whom one gets infected especially in high burden settings where transmission often takes place in the community and hence our proxy measure is unlikely to be a precise measure of recent infection. The positive CD8-mediated activity cut-off used in literature for comparison has not yet been fully established as standard cut-point [11]. Geographical variability may account for the differences noted as these studies were conducted in low or intermediate burden settings as compared to our high burden setting.

### Strengths and limitations

A strength of our study is that it based on a large randomly selected group of AYP in 14 communities in Zambia and Western Cape, South Africa which should have provided an unbiased estimate of the prevalence of infection among AYP in these communities. The study also provided a unique opportunity to examine antigen tube responses in the QFT-Plus assay and its associations.

Unlike in Zambia, where the HIV status of all participants could be confirmed a large proportion (39.0%) of participants in our South African cohort declined to test at the field site and did not consent for laboratory testing and so their HIV status was unknown. This limited our ability to fully examine the associations between HIV positivity and QFT-Plus positivity in South Africa.

Another limitation of our study is that HHC data were self-reported and therefore we may not have been able to fully ascertain the level of household exposure.

## Conclusion

TB infection was associated with age, household contact and alcohol among the adolescents and young people in Zambia. There were no associations identified in South Africa. The high prevalence of infection in these 14 communities warrants urgent action to address TB control, especially in South Africa, to prevent progression to disease.

Further research is required to delineate precise TB1 and TB2 responses in stringent recent exposure scenarios if the full benefit of the additional TB2 tube of the QFT-Plus assay is to be realized in high TB HIV burden settings, where it is most needed.

## Supporting information

S1 Checklist STROBE statement—Checklist of items that should be included in reports of observational studies. (DOC)

S1 Table QFT-Plus positivity risk factor analysis-Zambia. (DOCX)

S2 Table QFT-Plus positivity risk factor analysis-South Africa. (DOCX)
