# Supplementary material for: Prevalence and risk factors of M tuberculosis infection in young people across 14 communities in Zambia and South Africa
Source: PLOS Glob Public Health. 2023 Sep 29;3(9):e0002077. doi: 10.1371/journal.pgph.0002077 (PMC10540968; doi:10.1371/journal.pgph.0002077)
Supplement: S1 Checklist — (DOC) [file pgph.0002077.s001.doc]

STROBE Statement—checklist of items that should be included in reports of observational studies

|  | Item No | Recommendation |
| --- | --- | --- |
| **Title and abstract** | 1 | (*a*) Indicate the study’s design with a commonly used term in the title or the abstract  [Abstract says “we describe the baseline prevalence and risk factor of Mycobacterium Tuberculosis infection among this cohort.”] |
|  |  | (*b*) Provide in the abstract an informative and balanced summary of what was done and what was found  [What was done is described in the first paragraph of the “Methods and Findings” section. What was found is described in the second paragraph of the “Methods and Findings” section.] |
| Introduction | | |
| Background/rationale | 2 | Explain the scientific background and rationale for the investigation being reported  [See introduction, paragraphs 1-3.] |
| Objectives | 3 | State specific objectives, including any prespecified hypotheses  [See introduction, paragraph 3, and methods paragraph 4.] |
| Methods | | |
| Study design | 4 | Present key elements of study design early in the paper  [Provided in paragraph 1 of Methods, section “ Study design and setting ”.] |
| Setting | 5 | Describe the setting, locations, and relevant dates, including periods of recruitment, exposure, follow-up, and data collection  [Provided in paragraph 1 of Methods, section “Study design and setting”.] |
| Participants | 6 | (*a*) *Cohort study*—Give the eligibility criteria, and the sources and methods of selection of participants. Describe methods of follow-up  *Case-control study*—Give the eligibility criteria, and the sources and methods of case ascertainment and control selection. Give the rationale for the choice of cases and controls  *Cross-sectional study*—Give the eligibility criteria, and the sources and methods of selection of participants  [Provided in paragraphs 1-3 of section “Study design and setting ”, paragraph 1 of Methods section “Study procedures”] |
|  |  | (*b*) *Cohort study*—For matched studies, give matching criteria and number of exposed and unexposed  *Case-control study*—For matched studies, give matching criteria and the number of controls per case  [Not applicable] |
| Variables | 7 | Clearly define all outcomes, exposures, predictors, potential confounders, and effect modifiers. Give diagnostic criteria, if applicable  [Provided in Methods, section “Statistical analysis”, paragraphs 1-4.] |
| Data sources/ measurement | 8* | For each variable of interest, give sources of data and details of methods of assessment (measurement). Describe comparability of assessment methods if there is more than one group  [Provided in Methods, section “paragraph 1 and Methods, section “Statistical analysis”, paragraphs 1-4 ] |
| Bias | 9 | Describe any efforts to address potential sources of bias  [Provided in Methods, section “Statistical analysis”, paragraphs 1-4.] |
| Study size | 10 | Explain how the study size was arrived at  [Provided in Methods, section “Statistical analysis”, paragraphs 2 and Fig 1 gives the flow chart of the study participants. Analyses in this manuscript were based on the baseline measurement of infection and to participants with a valid QFT-Plus result.] |
| Quantitative variables | 11 | Explain how quantitative variables were handled in the analyses. If applicable, describe which groupings were chosen and why  [Provided in Methods, section “Statistical analysis”, paragraphs 1-4.] |
| Statistical methods | 12 | (*a*) Describe all statistical methods, including those used to control for confounding  [Provided in Methods, section “Statistical analysis”, paragraphs 1-4.] |
|  |  | (*b*) Describe any methods used to examine subgroups and interactions  [Provided in Methods, section “Statistical analysis”, paragraphs 1-4.] |
|  |  | (*c*) Explain how missing data were addressed  [Provided in Methods, section “Statistical analysis”, paragraphs 1-4.] |
|  |  | (*d*) *Cohort study*—If applicable, explain how loss to follow-up was addressed  *Case-control study*—If applicable, explain how matching of cases and controls was addressed  *Cross-sectional study*—If applicable, describe analytical methods taking account of sampling strategy  [Provided in Methods, section “Statistical analysis”, paragraphs 1-4.] |
|  |  | (*e*) Describe any sensitivity analyses  [Not applicable.] |

Continued on next page

| Results | | |
| --- | --- | --- |
| Participants | 13* | (a) Report numbers of individuals at each stage of study—eg numbers potentially eligible, examined for eligibility, confirmed eligible, included in the study, completing follow-up, and analysed  [Provided in Results, paragraph 1-2 , and Fig 1] |
|  |  | (b) Give reasons for non-participation at each stage  [Provided in Fig 1.] |
|  |  | (c) Consider use of a flow diagram  [Provided in Fig 1.] |
| Descriptive data | 14* | (a) Give characteristics of study participants (eg demographic, clinical, social) and information on exposures and potential confounders  [Provided in Table 1-3 and Supplementary Table S1a-S1b, and Supplementary Table S2] |
|  |  | (b) Indicate number of participants with missing data for each variable of interest  [Provided in Table 1-3 and Supplementary Table S1a-S1b, and Supplementary Table S2] |
|  |  | (c) *Cohort study*—Summarise follow-up time (eg, average and total amount)  [Not applicable] |
| Outcome data | 15* | *Cohort study*—Report numbers of outcome events or summary measures over time |
|  |  | *Case-control study—*Report numbers in each exposure category, or summary measures of exposure |
|  |  | *Cross-sectional study—*Report numbers of outcome events or summary measures  [Data on the numbers with the outcome, and summary measures, are provided in the Results text, tables, and figures.] |
| Main results | 16 | (*a*) Give unadjusted estimates and, if applicable, confounder-adjusted estimates and their precision (eg, 95% confidence interval). Make clear which confounders were adjusted for and why they were included  [Results for the key variables of interest are provided throughout the Results text, and in Table 1-3 and Supplementary Table S1a-S1b ] |
|  |  | (*b*) Report category boundaries when continuous variables were categorized  [Not applicable] |
|  |  | (*c*) If relevant, consider translating estimates of relative risk into absolute risk for a meaningful time period  [Not applicable.] |
| Other analyses | 17 | Report other analyses done—eg analyses of subgroups and interactions, and sensitivity analyses  [Analysis of social mixing pattern shown in Supplementary Table S2.] |
| Discussion | | |
| Key results | 18 | Summarise key results with reference to study objectives  [Provided in Discussion, paragraph 1-4] |
| Limitations | 19 | Discuss limitations of the study, taking into account sources of potential bias or imprecision. Discuss both direction and magnitude of any potential bias  [Provided in Discussion, paragraph 5] |
| Interpretation | 20 | Give a cautious overall interpretation of results considering objectives, limitations, multiplicity of analyses, results from similar studies, and other relevant evidence  [Provided in Discussion, paragraphs 2- 8] |
| Generalisability | 21 | Discuss the generalisability (external validity) of the study results  [Provided in Discussion, paragraph 6 -7 and the Conclusion] |
| Other information | | |
| Funding | 22 | Give the source of funding and the role of the funders for the present study and, if applicable, for the original study on which the present article is based  [A statement on this was provided as part of the manuscript submission] |

*Give information separately for cases and controls in case-control studies and, if applicable, for exposed and unexposed groups in cohort and cross-sectional studies.

**Note:** An Explanation and Elaboration article discusses each checklist item and gives methodological background and published examples of transparent reporting. The STROBE checklist is best used in conjunction with this article (freely available on the Web sites of PLoS Medicine at http://www.plosmedicine.org/, Annals of Internal Medicine at http://www.annals.org/, and Epidemiology at http://www.epidem.com/). Information on the STROBE Initiative is available at www.strobe-statement.org.
