# Supplementary material for: Prevalence and risk factors of M tuberculosis infection in young people across 14 communities in Zambia and South Africa
Source: PLOS Glob Public Health. 2023 Sep 29;3(9):e0002077. doi: 10.1371/journal.pgph.0002077 (PMC10540968; doi:10.1371/journal.pgph.0002077)
Supplement: S1 Table — (DOCX) [file pgph.0002077.s002.docx]

**S1 Table: QFT-Plus positivity risk factor analysis -Zambia***

| **Zambia** | | | | | | | | |
| --- | --- | --- | --- | --- | --- | --- | --- | --- |
| **Characteristic** | **N** | **%**  **Column** | **QFT plus Positive** | **%**  **(row)** | **AOR 1**  **(95%CI)** | **p** | **AOR 2^Z^**  **(95%CI)** | **p** |
| **Community of residence** |  |  |  |  |  | <0.001 |  | <0.001 |
| Z2 | 333 | 13.1% | 89 | 26.7% | 1 |  | 1 |  |
| Z3 | 346 | 13.6% | 101 | 29.2% | 1.11 (0.73-1.67) |  | 1.05 (0.70-1.58) |  |
| Z4 | 326 | 12.8% | 135 | 41.1% | 2.10 (1.40-3.15) |  | 1.82 (1.21-2.73) |  |
| Z5 | 324 | 12.7% | 117 | 36.1% | 1.67 (1.09-2.47) |  | 1.68 (1.12-2.50) |  |
| Z7 | 294 | 11.5% | 137 | 46.6% | 2.64 (1.74-4.01) |  | 2.40 (1.59-3.63) |  |
| Z8 | 326 | 12.8% | 152 | 46.6% | 2.91 (1.93-4.48) |  | 2.74 (1.83-4.10) |  |
| Z10 | 310 | 12.2% | 85 | 27.4% | 1.06 (0.70-1.62) |  | 0.92 (0.61-1.41) |  |
| Z12 | 293 | 11.5% | 60 | 20.5% | 0.70 (0.45-1.09) |  | 0.69 (0.44-1.07) |  |
| **Household Density**** |  |  |  |  |  | 0.578 |  | 0.519 |
| <1.2 | 463 | 18.1% | 157 | 33.9% | 1 |  | 1 |  |
| >1.2-1.67 | 878 | 34.4% | 286 | 32.6% | 0.92 (0.69-1.21) |  | 0.91 (0.68-1.20) |  |
| >1.67-2.33 | 582 | 22.8% | 193 | 31.2% | 0.90 (0.66-1.23) |  | 0.88 (0.66-1.20) |  |
| >2.33 | 629 | 24.7% | 240 | 38.2% | 1.08 (0.79-1.46) |  | 1.07 (0.78-1.45) |  |
| **Number of people sharing a room** |  |  |  |  |  | 0.899 |  | 0.984 |
| No sharing | 204 | 8.0% | 70 | 34.3% | 1 |  | 1 |  |
| 1 | 578 | 22.6% | 197 | 34.1% | 1.12 (0.75-1.67) |  | 1.12 (0.76-1.67) |  |
| 2 | 832 | 32.6% | 279 | 33.5% | 1.09 (0.74-1.61) |  | 1.05 (0.72-1.56) |  |
| 3 | 616 | 24.1% | 212 | 34.4% | 1.07 (0.72-1.59) |  | 1.05 (0.78-2.00) |  |
| 4 | 226 | 8.9% | 86 | 38.1% | 1.31 (0.82-2.11) |  | 1.25 (0.78-2.00) |  |
| 5 or more | 96 | 3.8% | 33 | 33.3% | 1.08 (0.58-2.00) |  | 1.03 (0.56-1.91) |  |

AOR 1 : Adjusted odds ratio : analysis adjusted for age, sex and community

AOR 1 : Adjusted odds ratio : analysis adjusted for age, sex and community

AOR 2^SA^: Adjusted odds ratio : analysis adjusted for age, sex and community, household contact, smoking, alcohol use and social mixing score

*Analysis excludes indeterminate QFT-plus results

** Household density: number of people / number of rooms
