# Supplementary material for: Prevalence and risk factors of M tuberculosis infection in young people across 14 communities in Zambia and South Africa
Source: PLOS Glob Public Health. 2023 Sep 29;3(9):e0002077. doi: 10.1371/journal.pgph.0002077 (PMC10540968; doi:10.1371/journal.pgph.0002077)
Supplement: S2 Table — (DOCX) [file pgph.0002077.s003.docx]

**S2 Table : QFT-Plus positivity risk factor analysis -South Africa***

| **Characteristic** | **N** | **%**  **Column** | **QFT plus Positive** | **%**  **(row)** | **AOR1**  **(95%CI)** | **p** | **AOR2^SA^**  **(95%CI)** | **p** |
| --- | --- | --- | --- | --- | --- | --- | --- | --- |
| **Community of residence** |  |  |  |  |  |  |  |  |
| SA14 | 321 | 16.2% | 230 | 71.7% | 1 | <0.001 | 1 | 0.003 |
| SA15 | 336 | 17.0% | 206 | 61.3% | 0.60 (0.41-0.87) |  | 0.62 (0.43-0.90) |  |
| SA16 | 329 | 16.6% | 203 | 61.7% | 0.64 (0.44-0.94) |  | 0.68 (0.47-1.00) |  |
| SA17 | 327 | 16.5% | 185 | 56.6% | 0.52 (0.34-0.76) |  | 0.58(0.40-0.85) |  |
| SA19 | 332 | 16.8% | 207 | 62.4% | 0.64 (0.44-0.93) |  | 0.62 (0.42-0.90) |  |
| SA21 | 332 | 16.8% | 249 | 75.0% | 1.21 (0.82-1.79) |  | 1.17 (0.79-1.74) |  |
| **Household Density**** |  |  |  |  |  | 0.105 |  | 0.165 |
| <1.2 | 515 | 26.1% | 323 | 62.7% | 1 |  | 1 |  |
| >1.2-1.67 | 554 | 28.0% | 355 | 64.1% | 1.09 (0.83-1.43) |  | 1.07 (0.81-1.40) |  |
| >1.67-2.33 | 331 | 16.7% | 214 | 64.7% | 1.18 (0.86-1.63) |  | 1.14 (0.83-1.57) |  |
| >2.33 | 577 | 29.2% | 338 | 67.2% | 1.42 (1.06-1.90) |  | 1.36 (1.02-1.82) |  |
| **Number of people sharing a room** |  |  |  |  |  | 0.352 |  | 0.702 |
| No sharing | 554 | 28.0% | 367 | 66.3% | 1 |  | 1 |  |
| 1 | 559 | 28.3% | 355 | 63.5% | 0.90 (0.69-1.19) |  | 0.94 (0.72-1.23) |  |
| 2 | 450 | 22.7% | 289 | 64.2% | 1.00 (0.74-1.35) |  | 1.04 (0.77-1.40) |  |
| 3 | 238 | 12.0% | 146 | 61.3% | 0.85 (0.60-1.21) |  | 0.91 (0.64-1.30) |  |
| 4 | 121 | 6.1% | 80 | 66.1% | 1.01 (0.64-1.61) |  | 1.05 (0.66-1.66) |  |
| 5 or more | 55 | 2.8% | 46 | 78.2% | 1.91 (0.92-3.95) |  | 1.82 (0.88-3.74) |  |

AOR 1 : Adjusted odds ratio : analysis adjusted for age, sex and community

AOR 2^SA^ : Adjusted odds ratio : analysis adjusted for age, sex and community, household contact, HIV status smoking, alcohol use and exposure score

*Analysis excludes indeterminate QFT-plus results

** Household density: number of people / number of room
